# New Remedy for Snake Bites and Rabies

**Published:** 1892-02

**Authors:** 


New Remedy for Snake Bites and Rabies.—The Berlin correspondent of the *Theraputic Gazette* says that a remedy for blood poisoning caused by the bites of snakes and rabid dogs has been discovered in Africa, by a Dr. Engels, in the "wild growing, black, noble palm." Five hundred negroes bitten by poisonous snakes were treated with the extract of the noble palm, and four hundred and eighty-seven were cured in five days. Of sixty-seven farmers and negroes bitten by rabid dogs sixty-five were saved, while two died of weakness. The remedy is injected under the skin, and causes a moderate fever, not exceeding 35.5 degs. C. On the third day the patient is without fever, swelling and inflammation of the affected part having disappeared, and on the fifth, or latest, on the seventh day, the patient is cured.—*Ex.*

